# Neuroimaging studies exploring the neural basis of social isolation

**DOI:** 10.1017/S2045796021000135

**Published:** 2021-04-06

**Authors:** Niccolò Zovetti, Maria Gloria Rossetti, Cinzia Perlini, Paolo Brambilla, Marcella Bellani

**Affiliations:** 1 Department of Neurosciences, Biomedicine and Movement Sciences, Section of Psychiatry, University of Verona, Verona, Italy; 2 Department of Neurosciences and Mental Health, Fondazione IRCCS Ca’ Granda Ospedale Maggiore Policlinico, Milan, Italy; 3 Department of Neurosciences, Biomedicine and Movement Sciences, Section of Clinical Psychology, University of Verona, Verona, Italy; 4 Department of Pathophysiology and Transplantation, University of Milan, Milan, Italy

**Keywords:** Social Brain, Social Isolation, Magnetic Resonance, Neurobiology

## Abstract

According to the social brain hypothesis, the human brain includes a network designed for the processing of social information. This network includes several brain regions that elaborate social cues, interactions and contexts, i.e. prefrontal paracingulate and parietal cortices, amygdala, temporal lobes and the posterior superior temporal sulcus. While current literature suggests the importance of this network from both a psychological and evolutionary perspective, little is known about its neurobiological bases. Specifically, only a paucity of studies explored the neural underpinnings of constructs that are ascribed to the social brain network functioning, i.e. objective social isolation and perceived loneliness. As such, this review aimed to overview neuroimaging studies that investigated social isolation in healthy subjects. Social isolation correlated with both structural and functional alterations within the social brain network and in other regions that seem to support mentalising and social processes (i.e. hippocampus, insula, ventral striatum and cerebellum). However, results are mixed possibly due to the heterogeneity of methods and study design. Future neuroimaging studies with longitudinal designs are needed to measure the effect of social isolation in experimental *v.* control groups and to explore its relationship with perceived loneliness, ultimately helping to clarify the neural correlates of the social brain.

The human nature is thought to be rooted in its social interactions and relationships, which play a crucial role in survival and reproduction and support the development and preservation of physical and mental health (Cacioppo *et al*., 2014). In humans, extreme cases of social isolation can lead to the complete avoidance of social contexts including work, education and those involving significant others such as friends or relatives. This condition is known as *Hikikomori syndrome* or complete withdrawal, a phenomenon that in 2013 was estimated to affect at least one million Japanese (Saito and Angles, 2013). The evidence to date shows that this phenomenon is growing in both eastern and western countries, possibly influenced by cultural and environmental factors such as parental style and urbanicity (Tateno *et al*., 2012) with severe consequences for the national health systems (Cacioppo and Cacioppo, 2018).

Specifically, social isolation (and related loneliness) has been associated with increased mortality risk and depressive symptomatology, poorer cognitive performance and executive functioning, faster cognitive decline, increased threat sensitivity and alterations in the cardiovascular and neuroendocrine systems in healthy individuals (Cacioppo and Hawkley, 2009; Bhatti and ul Haq, 2017).

Conversely, abundant social relationships and interactions were shown to exert a protective effect against dementia, neurocognitive decline and mortality. More importantly, an active social life has been found to correlate with healthy behaviours such as adequate sleep, diet and exercise and discourage unhealthy behaviours such as excessive eating (Kiely *et al*., 2000; House, 2001). While the study of social isolation and loneliness from a clinical perspective is important for clinicians and psychiatrists, the investigation of their neuronal correlates is also crucial as it may help to understand the neurobiological phenotype of the Hikikomori syndrome and other psychiatric disorders of which social isolation and loneliness are core symptoms (e.g. depression and schizophrenia). Therefore, we aimed to describe neurobiological correlates of social isolation by means of neuroimaging studies in healthy controls. Due to the paucity of studies measuring social isolation with quantifiable methods (i.e. confinement), we also included the studies that explored the neurobiological correlates of (self-reported) loneliness as the latter is considered a psychological correlate of objective social isolation (Cacioppo and Cacioppo, 2018).

The data search was conducted through PubMed, Scopus and Web of Science databases. The following keywords were used for the search: (‘neuroimaging’ OR ‘magnetic resonance imaging’ OR ‘electroencephalography’ OR ‘Positron Emission Tomography’ OR ‘diffusion tensor imaging’) AND (‘social isolation’ OR ‘loneliness’ OR ‘hikikomori’ OR ‘complete withdrawal’). The inclusion criteria were: (i) original articles published in peer-reviewed journals between January 2000 and October 2020; (ii) English language; (iii) the inclusion of healthy participants with no psychiatric or neurologic conditions; and (iv) the application of neuroimaging technique (i.e. magnetic resonance imaging (MRI), electroencephalography (EEG), diffusion tensor imaging (DTI), positron emission tomography (PET)). Preclinical studies and case-report were excluded. A flow diagram illustrating the studies selection process is presented in Fig. 1. The literature search retrieved 182 records. After title and abstract screening, 162 articles were excluded because they clearly did not meet inclusion criteria. The remaining 20 studies were included in this review after a full-text review. Sample characteristics and neuroimaging findings from each study are shown in Table 1.

**Fig. 1.**
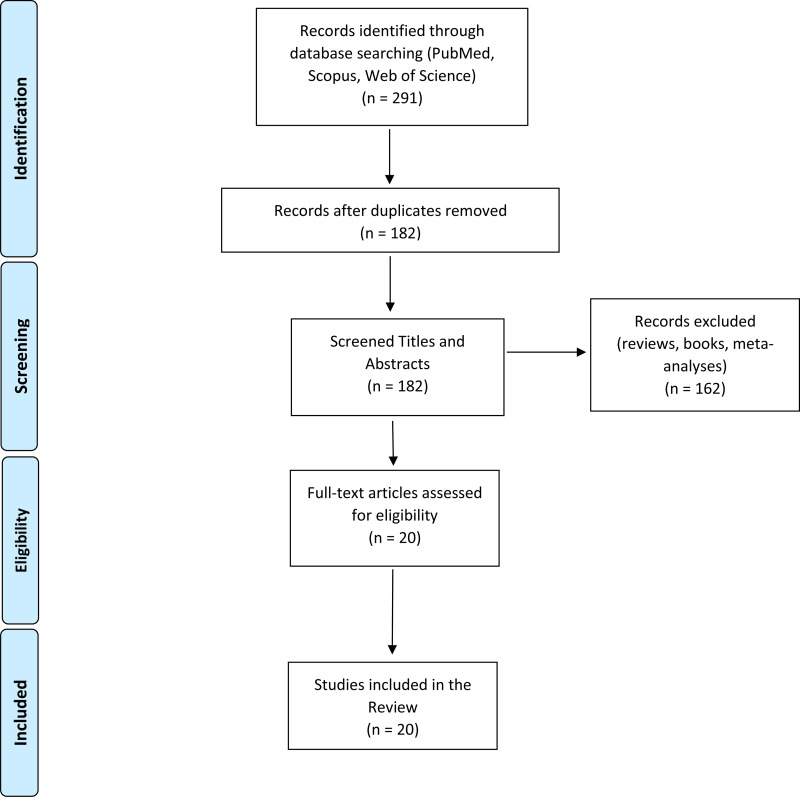
A flow diagram of the articles screening and selection process.

**Table 1.** Neuroimaging studies exploring the neurobiological correlates of objective social isolation and perceived loneliness

Reference	Study design	*N* participants (% *F*)	Age, mean (s.d.)	loneliness or social isolation assessment	Loneliness, mean (s.d.); range	Neuroimaging method	Results
Cacioppo *et al*. (2009)	Cross-sectional	23 (100%)	ns	UCLA-LS	ns	fMRI	*Participants with high perceived loneliness:* Increased activation of the ventral striatum when viewing pictures of people *vs.* objects (pleasant pictures) Decreased activation of the visual cortex when viewing pictures of people *vs.* objects (unpleasant pictures) *Participants with low perceived loneliness:* Increased activation of the bilateral temporoparietal junction when viewing pictures of people *vs.* objects
Kanai *et al*. (2012)	Cross-sectional	108 (57%)	23.5 (4.5)	UCLA-LS	ns	sMRI	Higher loneliness scores correlated with lower GM in the L posterior superior temporal sulcus
Powers *et al*. (2013)	Cross-sectional	34 (64%)	19.5 (ns)	Social exclusion manipulated experimentally	ns	fMRI	Social exclusion was associated with reduced activity in the dorsomedial PFC when viewing negative social scenes
Kong *et al*. (2015)	Cross-sectional	308 (54%)	19.9 (1.2)	UCLA-LS, EPQ	41.7 (7.9); 24–62	sMRI	Individuals with higher loneliness scores had increased GM volume in the L dorsolateral PFC
Nakagawa *et al*. (2015)	Cross-sectional	776 (55%)	20.7 (1.8)	UCLA-LS	37.0 (9.2); ns	DTI	Higher loneliness scores correlated with lower WM density in the bilateral inferior parietal lobule, R anterior insula, posterior temporoparietal junction, L posterior/superior temporal sulcus, dorsomedial PFC and rostrolateral prefrontal cortex
Jacubowski *et al*. (2015)	Longitudinal	6 (0%)	31.3 (4.1)	Confinement (18 months)	ns	EEG	After 18 months of social isolation, participants showed decreased global cortical activity, in both *α* and *β* activity
Cacioppo *et al*. (2016)	Cross-sectional	27 (55%)	24 (ns)	UCLA-LS	42.2 (11.2); 23–60	EEG	Participants with *vs.* without perceived loneliness differentiated more quickly social threat images from non-social threat stimuli In participants with high perceived loneliness, social threat images *v.* non-social threat stimuli evoked greater activations in the PFC, associative visual cortex, inferior and superior temporal gyrus, para-hippocampus, supramarginal gyrus, dorsolateral PFC cortex, amygdala
Donovan *et al*. (2016)	Cross-sectional	79 (60%)	76.4 (6.2)	UCLA-3-LS	5.3 (1.8); 3–10	PiB-PET	Results indicated an association between loneliness and cortical amyloid burden in elderly adults without cognitive deficits
Inagaki *et al*. (2016)	Cross-sectional	31 (48%)	24.2 (7.5)	UCLA-LS	44.2 (8.6); 33–69	fMRI	Loneliness was associated with greater activity in the ventral striatum while viewing pictures with a social valence *v.* neutral picture
Layden *et al*. (2017)	Cross-sectional	55 (56%)	23.7 (2.1)	UCLA-LS	40 (8.1); ns	fMRI	Higher perceived social isolation correlated with (i) greater activity in the R central operculum, R supramarginal gyrus; (ii) increased connectivity within the cingulo-opercular network, and (iii) reduced connectivity between the cingulo-opercular network and R middle/superior frontal gyrus
Tian *et al*. (2017)	Cross-sectional	30 (0%)	21.3 (2.4)	UCLA-LS	ns	fMRI	Higher loneliness was associated with (i) decreased blood flow from the dorsal to the ventral attentional network and from the affective to the visual network
Yi *et al*. (2018)	Cross-sectional	100 (46%)	28.5 (NS)	UCLA-LS	50.5 (8.6); ns	fMRI	Higher loneliness was associated with higher amplitudes of low-frequency fluctuations activity in the inferior temporal gyrus
Uquillas *et al*. (2018)	Cross-sectional	117 (69%)	76 (6.2)	UCLA-3-LS	5.19 (1.95); 3–12	PET	Higher loneliness scores were associated with higher tau pathology in the R entorhinal cortex and R fusiform gyrus.
D'Agostino *et al*. (2019)	Cross-sectional	50 (52%)	62.9 (6.1)	UCLA-LS	43.5 (8.9); ns	fMRI	No association between loneliness scores and ventral striatum and amygdala activity were found
Düzel *et al*. (2019)	Cross-sectional	319 (51%)	70.1 (3.7)	UCLA-LS	1.5 (0.6); ns	sMRI	Results showed a negative association between loneliness scores and GM volumes in the L amygdala, L anterior hippocampus, L posterior para-hippocampus and L cerebellum
Weber *et al*. (2019)	Longitudinal	33 (43%) (16 isolated; 17 not isolated)	36.3 (7.2)	Confinement (30 days)	ns	EEG	Isolation did not affect cognitive performance nor BDNF factor or mood when comparing participants with *vs.* without isolation, but EEG revealed the presence of decreased activity in the parietal cortex
Feng *et al*. (2019)	Cross-sectional	75 (13%)	21.8 (3)	UCLA-LS	ns	fMRI	Applying a machine learning algorithm, participants' loneliness scores were predicted by within- and between-network connectivity of prefrontal, limbic and temporal systems (i.e. dorsolateral PFC, lateral orbitofrontal cortex, ventromedial PFC, caudate, amygdala)
Mwilambwe-Tshilobo *et al*. (2019)	Cross-sectional	942 (53%)	28 (3.4)	Loneliness survey	50.9 (8.5); 37–82	fMRI	Loneliness was associated with fewer modular, connections between default, frontoparietal, attention and perceptual networks
Wong *et al*. (2019)	Cross-sectional	99 (51%)	28.6 (17.7)	UCLA-LS; Lubben social network scale	43 (7.7); ns	sMRI/fMRI	Greater posterior and medial cerebellar volumes in participants susceptible to loneliness *vs.* non-susceptible Posterior and medial cerebellar functional activations when processing positive *vs.* neutral words exhibited significant interactive effects of both loneliness and social network Loneliness predicted the R posterior cerebellum functional connectivity with the R visual cortex Social network size predicted R posterior cerebellum functional connectivity with the R premotor cortex during the processing of positive words
Stahn *et al*. (2019)	Longitudinal	18 (44%) (9 isolated; 9 not isolated)	(ns)	Polar expedition (18 months)	ns	sMRI	After 18 months, isolated participants showed reduced GM volumes of the hippocampus (dentate gyrus)

BDNF, brain-derived neurotrophic factor; EEG, electroencephalogram; EPQ, Eysenck Personality Questionnaire; fMRI, functional magnetic resonance imaging; GM, grey matter; L, left; ns, not specified; PET, positron emission tomography; PFC, prefrontal cortex; PiB, Pittsburgh compound B; R, right; sMRI, structural magnetic resonance imaging; UCLA-LS, The University of California Los Angeles, Loneliness Scale; UCLA-3-LS, 3-item version UCLA-LS; vs., versus; WM, white matter.

Seventy per cent of the studies (14 out of 20) explored the neurofunctional correlates of social isolation or loneliness with either fMRI, EEG or PET, while the remaining 30% investigated the neurostructural correlates of those constructs (i.e. grey matter (GM) volumes and white matter (WM) integrity) through structural MRI and DTI.

The University of California Los Angeles Loneliness Scale (UCLA-LS) was used in 16 out of the 20 studies (76%), to measure the feeling of loneliness. The UCLA-LS is a self-report instrument and consists of 20 items measuring general loneliness and satisfaction with social relationships. Participants are instructed to indicate how often they feel the way described by each item on a Likert scale (1 = never, 2 = rarely, 3 = sometimes, 4 = always).

As shown in Table 1, the extent of loneliness varied across studies. Specifically, the mean UCLA score was available in 12 out of 20 studies and ranged between 23 and 69 corresponding, respectively, to a low-to-high degree of loneliness, with the mean UCLA values ranging between 40 and 44 (moderate loneliness). Other studies however did not report the loneliness scores of their samples or measured objective isolation instead (confinements, polar expeditions).

Studies investigating the association between loneliness and structural brain correlates showed mixed findings. Specifically, higher loneliness scores correlated with reduced GM volumes in the posterior superior temporal sulcus, amygdala, hippocampus/para-hippocampus and cerebellum (Kanai *et al*., 2012; Düzel *et al*., 2019); increased GM volumes in the dorsolateral prefrontal cortex (PFC) (Kong *et al*., 2015); and lower WM density in the bilateral inferior parietal lobule, right insula, posterior temporoparietal junction, posterior superior temporal sulcus, dorsomedial and rostro-lateral PFC (Nakagawa *et al*., 2015). Moreover, increased GM cerebellar volumes were observed when comparing individuals with *v.* without susceptibility to loneliness (Wong *et al*., 2019). Lastly, Stahn *et al*. (2019) conducted a longitudinal study to explore the effects of prolonged isolation on structural brain indices and found that after 14 months of isolation, the experimental group (i.e. polar expeditioners) showed lower hippocampal volumes compared to a control group matched by age, sex and baseline hippocampal volume (Stahn *et al*., 2019).

Studies investigating the association between loneliness and functional brain correlates analysed the blood oxygenation level-dependant (BOLD) or EEG signals of the samples. Overall, loneliness was associated with different processing of pleasant and unpleasant social stimuli and words with affective value (Cacioppo *et al*., 2009, 2016; Inagaki *et al*., 2016; Wong *et al*., 2019). Specifically, Cacioppo *et al.* showed that, while viewing pleasant and unpleasant pictures, participants with high *v.* low perceived loneliness (as measured by the UCLA-LS) had reduced activations of the ventral striatum and the temporoparietal junction and greater activation of the visual cortex (Cacioppo *et al*., 2009). The same authors found also that images depicting social threat stimuli were differentiated more quickly by individuals with higher *v.* lower loneliness scores and were associated with greater activations in the PFC, visual cortex, temporal gyrus, hippocampus and supramarginal gyrus, only in lonely individuals (Cacioppo *et al*., 2016). Similarly, Wong *et al*. (2019) observed that loneliness level was positively associated with the right posterior cerebellar functional connectivity with the visual and premotor cortices when processing words with positive *v.* neutral affective value (Wong *et al*., 2019). Lastly, Powers *et al*. (2013) manipulated experimentally the feeling of social exclusion perceived by the participants by asking them to complete a personality questionnaire and providing them with false feedback randomly assigned. Then, participants underwent an fMRI exam while viewing pictures with affective value. Participants in which social exclusion was experimentally induced showed lower PFC activations when viewing negative social scenes (Powers *et al*., 2013). Conversely, D'Agostino *et al*. (2019) found no association between loneliness scores and brain activations during an fMRI task showing participants pictures depicting pleasant social stimuli of strangers (D'Agostino *et al*., 2019). This finding contrasted with previous studies reporting decreased ventral striatum activity as a function of loneliness during a similar task (Cacioppo *et al*., 2009). The authors suggested that discrepancies with previous studies and the absence of any association between loneliness and different processing of social cues might have been due to differences in sample size and stimuli presented.

Other studies examined the BOLD activity of the brain at rest through resting-state functional MRI and showed that social isolation and perceived loneliness were associated with increased activity in the right central operculum, right supramarginal gyrus and between default, frontoparietal, attention and perceptual networks (Layden *et al*., 2017; Mwilambwe-Tshilobo *et al*., 2019). Two studies found that higher loneliness scores were associated with (i) decreased blood flow from the dorsal to the ventral attentional network, (ii) decreased flow from the affective to the visual network and (iii) low-frequency fluctuations of the activity in the inferior temporal gyrus (Tian *et al*., 2017; Yi *et al*., 2018). In line with these results, Feng *et al*. (2019) applied a machine learning discrimination algorithm to the resting-state activations of healthy participants and showed that individual loneliness scores could be predicted by the connectivity of the prefrontal, limbic and temporal networks (Feng *et al*., 2019). Specifically, key nodes contributing to the discrimination process were in the PFC, lateral orbitofrontal cortex (OFC), caudate, amygdala and temporal regions.

Finally, prolonged objective isolation (i.e. confinement) was associated with decreased global and parietal cortical activity when measured through EEG (Jacubowski *et al*., 2015; Weber *et al*., 2019). Specifically, Jacubowski *et al*. (2015) evaluated the impact of the stress caused by isolation in a group of subjects that lived in confinement for 520 days. While isolated, participants were instructed to engage in physical training for 30 min/day. EEG data were collected before and after each exercise session every 2 weeks, cortisol was collected every 2 months. Results indicated decreased global cortical activity (i.e. *α* and *β* activity) and increased salivary cortisol level throughout the isolation period, thus indicating a potential stressful effect of isolation on the brain (Jacubowski *et al*., 2015).

Lastly, only two studies investigated the neurobiological correlates of loneliness through PET imaging and found that perceived loneliness was associated with increased amyloid and tau proteins, aggregates commonly associated with the development of several neurocognitive diseases (Donovan *et al*., 2016; Uquillas *et al*., 2018).

To summarise, current literature indicates that perceived loneliness and objective social isolation were associated with structural and functional changes in several brain regions including prefrontal, temporal and parietal cortices, limbic structures (i.e. hippocampus, amygdala, insula), the striatum and the cerebellum. Part of these brain regions belongs to the *social brain network*, a complex neural circuit that elaborates social cues, interactions and contexts (Dunbar, 2009). Specifically, the network encompasses the OFC, the medial PFC, the para-cingulate cortices, the amygdala, the temporal lobes and the posterior superior temporal sulcus (Frith, 2007; Adolphs, 2009).

The OFC and medial PFC are known to be involved in the monitoring of social behaviours and mentalising processes (Beer *et al*., 2006). For instance, patients with structural OFC alterations show a poor control of emotions and decision-making processes (Bellani and Brambilla, 2008). Similarly, the medial PFC seems to be involved in the understanding and decoding of others' behaviours in terms of mental states and social interactions (Iacoboni *et al*., 2004). Also, the anterior cingulate cortex has been suggested to play a role in the processing of social information (Apps *et al*., 2016). The role of the amygdala in social cognition is well known as its activity has been widely associated with the processing of emotions, prejudice, social judgements and behaviours (Adolphs and Spezio, 2006). Lastly, temporal lobes and the posterior superior temporal sulcus have been extensively studied in the context of the social brain as their alterations are frequently associated with autism spectrum disorders, a condition in which social cognition is severely compromised (Saitovitch *et al*., 2012). Particularly, the posterior superior temporal sulcus and the temporoparietal junction have been associated with the detection and processing of eye movements (Pelphrey *et al*., 2005).

Overall, our findings suggest that objective social isolation and perceived loneliness are associated with morpho-functional changes that extend both in and out of the social brain network (i.e. PFC, temporal sulcus, amygdala and temporal cortex, parietal cortex and hippocampus) (Fig. 2). Even though some of the brain regions associated with social isolation and perceived loneliness (e.g. parietal lobule and cortex, hippocampus, cerebellum) were not included in the early theorisation of the human social brain, recent evidence suggests that they also play a role in social cognition (Rizzolatti and Fabbri-Destro, 2008; Laurita and Spreng, 2017). For example, the parietal cortex is home to the mirror neurons system, a complex network allowing primates and humans to understand the intention behind an observed motor act (Rizzolatti and Fabbri-Destro, 2008) while the hippocampus is not only involved in recalling and storing memories, but also in social navigation, namely the formation of dynamical social relationships including social distances, hierarchies and social bonds (Laurita and Spreng, 2017). Lastly, the cerebellum has been suggested to provide a supportive role in social cognition and in higher abstraction mentalising processes (Van Overwalle *et al*., 2014). As such, it could be speculated that social cognition (and related disorders) relies on a diffused brain circuit, broader than the social brain network, in which different regions play a central or supplementary role (Bellani and Brambilla, 2008; Crippa *et al.*, 2016). However, results are still sparse and need replication.

**Fig. 2.**
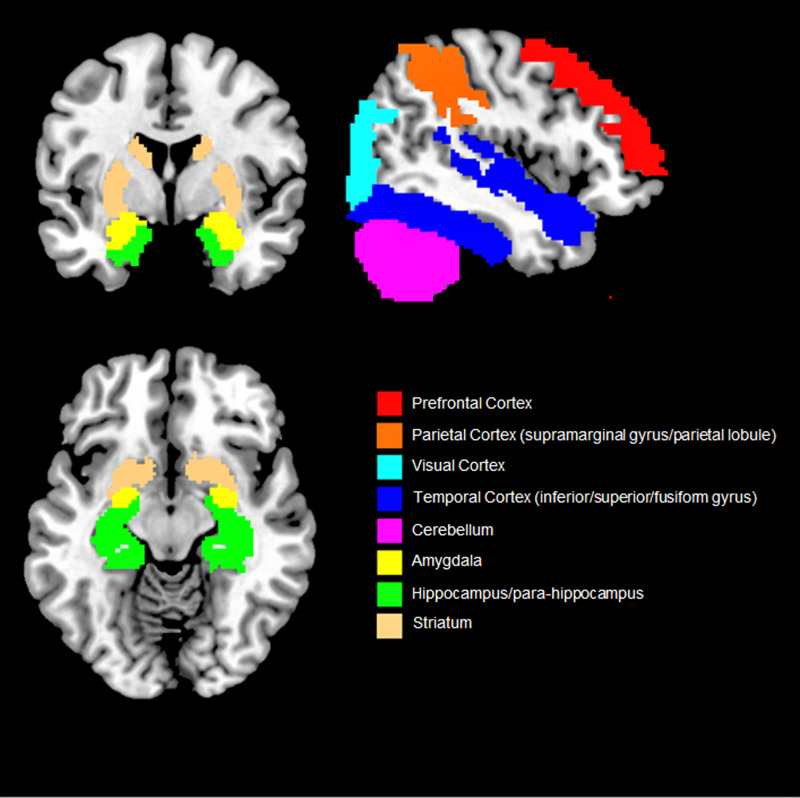
Overview of the brain regions more consistently found associated with social isolation and loneliness. Only results that were reported by two or more studies are shown.

A number of limitations should be considered when interpreting the results. First, 85% of the studies were cross-sectional and measured the correlation between self-reported loneliness and brain indices while only three studies had a longitudinal design and could measure the effect of objective isolation on the brain. Moreover, the magnitude and the precision of the observed effects remain ambiguous as none of the studies reported the effect size or the confidence intervals of the results. This, in turn, made it difficult to compare the studies with each other, precluding the generalisability of the findings. Therefore, this review highlights the need to conduct future neuroimaging studies by using longitudinal designs and more quantifiable measures of social isolation (e.g. confinement). This could help clarifying the neurobiological signature of the Hikikomori syndrome and other psychiatric disorders of which social isolation and loneliness are core symptoms (e.g. depression and schizophrenia) and, ultimately, inform future diagnostic system and tailored treatments.

## Data Availability

The data that support the findings of this study (search query) are available on request from the corresponding author.
